# Mortality and associated risk factors in a cohort of tuberculosis patients treated under DOTS programme in Addis Ababa, Ethiopia

**DOI:** 10.1186/1471-2334-11-127

**Published:** 2011-05-16

**Authors:** Belete Getahun, Gobena Ameni, Sibhatu Biadgilign, Girmay Medhin

**Affiliations:** 1 Aklilu Lemma Institute of Pathobiology, College of Health Sciences Addis Ababa University, PO Box 1176, Addis Ababa, Ethiopia; 2 Armauer Hansen Research Institute, PO Box 1005, Addis Ababa, Ethiopia; 3 Department of Epidemiology and Biostatistics, Jimma University, College of Public Health and Medical Science, Ethiopia P.O.Box 24414, Addis Ababa, Ethiopia

**Keywords:** Mortality, cohort, tuberculosis, DOTS, Ethiopia

## Abstract

**Background:**

Tuberculosis (TB) is the leading cause of mortality among infectious diseases worldwide. Ninty five percent of TB cases and 98% of deaths due to TB occur in developing countries. Globally, the mortality rate has declined with the introduction of effective anti TB chemotherapy. Nevertheless, some patients with active TB still die while on treatment for their disease. In Ethiopia, little is known on survival and risk factors for mortality among a cohort of TB patients. The objective of the study is to determine the magnitude and identify risk factors associated with time to death among TB patients treated under DOTS programme in Addis Ababa, Ethiopia.

**Methods:**

This is a retrospective cohort study. Data was obtained by assessing medical records of TB patients registered from June 2004 to July 2009 G.C and treated under the DOTS strategy in three randomly selected health centers. A step-wise multivariable Cox's regression model and Kaplan- Meier curves were used to model the outcome of interest. Mortality was used as an outcome measure. Person-years of observation (PYO) were calculated from the date of starting anti-TB treatment to date of outcome and was calculated as the number of deaths/100 PYO. Statistical analysis SPSS version 16 was used for data analysis and results were reported significant whenever P-value was less than 5%.

**Results:**

From a total of 6,450 registered TB patients 236(3.7%) were died. More than 75% death occurred within eight month of treatment initiation. The mean and median times of survival starting from the date of treatment initiation were 7.2 and 7.9 months, respectively. Comparison of survival curves using Kaplan Meier curves method with log-rank test showed that the survival status was significantly different between patient categories as well as across treatment centers (P < 0.05). The death rate of pulmonary positive, pulmonary negative and extra pulmonary TB patients were 2.7%, 3.6%, and 4.3%, respectively. Body weight at initiation of anti-TB treatment (<35 kg), patient category, year of enrollment and treatment center were independent predictors for time to death.

**Conclusions:**

Most of the patients were died at the end of treatment period. This underlines the need for devising a mechanism of standardizing the existing DOTS programme and nutritional support for underweight patients for better clinical and treatment outcome.

## Background

In 2008, there were an estimated 8.9-9.9 million incident cases of TB, 9.6-13.3 million prevalent cases of TB, an estimated 1.3 million (range, 1.1-1.7 million) deaths, including 0.5 million (range, 0.45-0.62 million) deaths among women, occurred among HIV-negative incident cases of TB, and there were an estimated 0.5 million deaths among incident TB cases who were HIV-positive [1]. In 2005, 15.4 million TB cases were reported globally, among 8.8 million people new TB cases, 3.9 million were smear positive, and 1.7 million people died of TB in the same year [2]. Ninety eight percent of TB deaths occur in the developing countries and if left unchecked, with in 20 years, TB will kill about 35 million people [2]. WHO developed the DOTS strategy as the internationally recommended approach to TB control in the mid-1990s. DOTS is also the foundation of the Stop TB Strategy, which was launched by WHO in 2006 to guide TB control efforts during the period 2006-2015. The start of WHO efforts to systematically monitor progress in TB control on an annual basis in 1995 coincided with global promotion and expansion of the DOTS strategy [1].

In Ethiopia, TB has long been recognized as a major public health problem since the 1950s [3] and the country has been implementing the WHO recommended DOTS (Directly Observed Treatment Short-course) strategy since 1992[4]. At present, TB control strategy in Ethiopia relies on WHO recommended Stop TB Strategy and it has been implemented in the country since 2006[5].According to the 2008 WHO TB report [4], Ethiopia ranks 7th in the list of the world's 22 high burden countries for TB with incidence estimated at 379/100,000 for all forms of TB and 168/100,000 for smear positive TB. According to the Ministry of Health hospital statistics data, TB mortality rate is estimated at 84 per 100,000 populations per year [3].

TB mortality has been variously attributed to a dramatic rise in drug resistance [6] and to deteriorations in TB services [7,8] and the risk of dying could be reduced through improved care [9,10]. So to avert the problem of TB mortality, policy makers should devise a plan, with clear reflection on the magnitude of mortality and associated risks factors of TB, to improve the quality and standard of TB care. The objective of this paper was to identify the magnitude and risk factors associated with death among a cohort of TB patients treated under DOTS programme in Addis Ababa, Ethiopia.

## Methods

### Study setting

This institution based retrospective study was carried out in three randomly selected health centers of Addis Ababa, the capital city of Ethiopia. Addis Ababa has a surface area of 540 Square kilometer. In the year 2007 the population of the city was about 2.74 million, 5046 people live per Square kilometer, and 52.4% of the residents were females [11]. Administratively, the city is divided into 10 sub-cities which are in turn divided into 99 lowest administrative units known as Kebeles. The health institutions in the city comprise of 5 district hospitals and 1 specialized hospital which are all administered by the Federal Ministry of Health (MOH), 5 hospitals administered by other Governmental Agencies and 30 private owned hospitals. Moreover, the city has 24 health centers run by the government. Similarly, in the private sector there are 109 special clinics, 169 higher clinics and 146 medium clinics. The Addis Ababa city administrations had potential health service coverage of 21% in 2009 G.C [12].

### Study design and participants

Health institution-based retrospective cohort study was conducted in Addis Ababa from February 2010 to March 2010 G.C to evaluate treatment outcome among TB patients. Information for this study was extracted from documents of all TB cases registered from June 2004 to July 2009 G.C in three DOTS clinic located in three randomly selected health centers. The three health centers constitute different health service coverage areas of the city. In order to attain representativeness of different health service coverage the sub cities were categorized by their health service coverage in to low, medium, and high health service coverage (Table 1) and the available health centers were listed in each group. From each health service coverage group one health center was randomly selected, namely, Teklehaymanot Health Center from high health service coverage, Selam Health Center from medium health service coverage, and Kolfe Heath Center from low health service converge.

**Table 1:** Health service coverage of Addis Ababa by sub cities, 2009.

*Sub cities*	*Health Service Coverage in%*	Remark*
Kolfe	16	Low
Nifas silk-Lafto	22	Low
Yeka	33	Low
Adisketema	35	Medium
Gullele	35	Medium
Bole	40	Medium
Arada	50	Medium
Kirkos	50	Medium
Akaki	60	High
Lideta	66	High

Addis Ababa	34.7	

Source: Addis Ababa City Administrative Health Bureau

*Working definition to enable us to stratify the sub cities.

### Definition of selected independent variables

#### Type of TB

**Figure 1:**
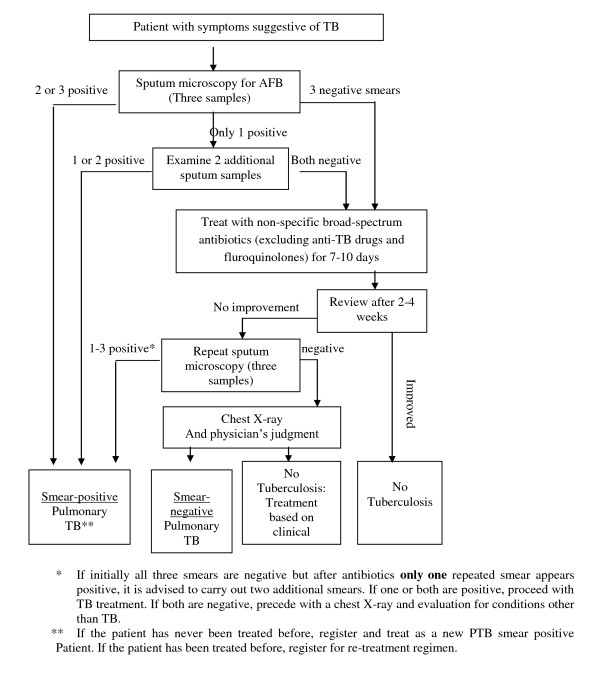
**Diagnostic algorithms for pulmonary TB and extra pulmonary TB (Adapted from the Federal Ministry of Health of Ethiopia (FMOH): Tuberculosis, Leprosy and TB/HIV Prevention and Control Programme Manual**. Addis Ababa: MOH 4th edition. 2008).

According to the standard definitions of the Ethiopian National Tuberculosis and Leprosy control Program guideline (NLCP)[5], type of TB was defined as (a) pulmonary TB, smear-positive (a patient with at least two sputum specimens which were positive for acid-fast bacilli (AFB) by microscopy, or a patient with only one sputum specimen which was positive for AFB by microscopy, and chest radiographic abnormalities consistent with active pulmonary TB), (b) pulmonary TB, smear-negative (a patient with symptoms suggestive of TB, with at least two sputum specimens which were negative for AFB by microscopy, and with chest radiographic abnormalities consistent with active pulmonary TB (including interstitial or miliary abnormal images), or a patient with two sets of at least two sputum specimens taken at least two weeks apart, and which were negative for AFB by microscopy, and radiographic abnormalities consistent with pulmonary TB and lack of clinical response to one week of broad spectrum antibiotic therapy) and (c) Extra pulmonary TB (EPTB) (this included tuberculosis of organs other than the lungs, such as lymph nodes, abdomen, genitourinary tract, skin, joints and bones, meninges, etc) [Figure 1].

#### Patient category

At entry to DOTS patients were categorized according to the Ethiopian NLCP national guideline (a) as new cases (patient who has never had treatment for TB before or has been on anti- TB treatment for less than four weeks ), (b) relapse (a patient who has been declared cure or treatment completed of any form of TB in the past but who reports back to the health service and is found to be AFB smear positive or culture positive), (c) treatment failure (a patient who, while on treatment remained smear- positive or become again smear-positive at the end of the five month or later after commencing treatment), (d) returned after default (a patient who had previously been recorded as defaulted from treatment and returns to the health facility and found to be smear positive sputum), (e) transfer in (a patient who started treatment in one health facility and transferred to another health facility to continue treatment), and (f) Other (a patient who does not fit in any of the above mentioned categories)

#### Treatment Outcome

this variable was recorded as (a) cured (finished treatment with negative bacteriology result at the end of treatment), (b) treatment completed (patient finished treatment but no bacteriology result at the end of the treatment), (c) treatment failure (smear positive at five months despite correct intake of medication), defaulter (patients who interrupted their treatment for two consecutive months or more after registration), died (patients who died from any cause during the course of treatment), (d) transferred out (patients whose treatment results are unknown due to transfer to another health facility) and (e) successfully treated (the sum of patients who are declared "cured" and those who have "completed" treatment). Survival time was defined as the time in days from the beginning of treatment to death from tuberculosis as the main or associated cause. Censoring occurred either at the end of the study or due to death from other causes and include defaulters and transfer outs.

### Definition for health service coverage

**Low health service coverage**: when the health service coverage is less than 33% for the sub city population.

**Medium health service coverage**: when the health service coverage is between 35-50% for the sub city population.

**High health service coverage**: when the health service coverage is greater than 50% for the sub city population.

### Data collection procedures

The registration documents of each heath centers contain basic information such as patient's age, sex, address, weight, types of TB (as defined above), AFB smear result at base line, 2^nd^, 5^th ^and 7^th ^month, treatment regimen, treatment started date, treatment stopped date and treatment outcome (as defined above). Data were collected by structured data sheet from the selected health centers registration book. Data were extracted from the registration book by health professionals (nurse/health officer) working in TB clinic after attending one day orientation on the structured data sheet by the principal investigator. To ensure quality of the collected data the following measures were taken: (a) A one day training was given for data collectors before the start of data collection, (b) he overall activities of data extraction were monitored by the principal investigator, and there was strict supervision during data collection, (c) all completed data sheets were examined for completeness during data collection, (d) consistencies of the collected data were checked during analysis.

### Analysis variables

The independent variables considered for the analysis were age, sex, type of TB, patient category (new, relapse, failed, return after default, transfer in, and other); AFB smear result and treatment start and stopped date. The dependent (outcome) variable was time to death of a patient which was ascertained by calculating the difference between the date of initiation of anti-TB treatment and date of outcome. The difference was divided by 30 to determine time to an event in month.

### Ethical consideration

Before reviewing patient documents ethical clearance was obtained from Aklilu Lemma Institute of Pathobiology, Addis Ababa University (ALIPB AAU), Addis Ababa City Administrative Health Bureau and health offices of the three randomly selected sub-cities'. Official letter of co-operation from health offices of the three sub-cities were written to respective health facilities. In order to ensure confidentiality of the information, names or identification number of study participants were not included in the data sheet.

### Statistical analyses

The quantitative data extracted from the registration book were checked for completeness and consistency by the principal investigator. Data entry and analyses were carried out using SPSS version 16. Descriptive statistical methods were used to summarize the socio-demographic characteristics of the study participants. Kaplan-Meier method with log rank test were used to estimate survival probability and compare survival curve for pulmonary positive, pulmonary negative and extra pulmonary TB patients. Cox's proportional hazards model was used to identify risk factors for time to death within treatment period using forward stepwise procedure. Variables showing significant association (P < 0.05) in the bivariate analysis were included in the multivariable Cox's proportional hazards model. Adjusted hazard ratio (AHR) and corresponding 95% confidence interval (95%CI) were estimated for potential risk factors included in the multivariable model. Mortality was used as an outcome measure. Person-years of observation (PYO) were calculated from the date of starting anti-TB treatment to date of outcome if the patient was alive, or to date of death. The study outcomes of participants were censored if they were reported to be dead at the time of data collection. Mortality was calculated as the number of deaths/100 PYO.

## Results

### Demographic characteristics

In this retrospective document analysis, socio-demographic and medical information of 6,450 registered TB patients was summarized. The mean, standard deviation and median age of the study participants were 30.13, 13.7 and 28.0 years, respectively. Out of the total study participants 46.8% were males and 77.9% were in the age group of 15-44 years. The study participates constitute were 46.5% from Teklehaymanot heath center, 16.7% from Selam heath center and 36.7% from Kolfe health centers. In total, 25.6% were pulmonary positives, 33.9% were pulmonary negatives and 40.5% were extra pulmonary TB patients. Majority of the study participants (88.9%) were new cases (Table 2).

**Table 2:** Characteristics of study participants in Addis Ababa, Ethiopia March 2010

Patient Characteristics	Number (%)
Sex	
Male	3017(46.77)
Female	3433(53.23)
Age categories	
0-14	459(7.12)
15-24	1932(29.95)
25-34	2023(31.36)
35-44	1069(16.57)
45-54	537(8.32)
55-64	251(3.89)
>65	179(2.77)
Treatment center	
Teklehaimanot HC	3002(46.54)
Selam HC	1078(16.71)
Kolfe HC	2370(36.75)
TB patients Category	
New	5736(88.93)
Relapse	160(2.48)
Failure	13(0.20)
Default	12(0.19)
Transfer in	164(2.54)
Others	365(5.66)
Type of TB	
Pul.postive	1652 (26.5)
Pul.negative	2187(33.9
Extra pulmonary	2611(40.5)

### Survival analysis of TB patients

**Figure 2:**
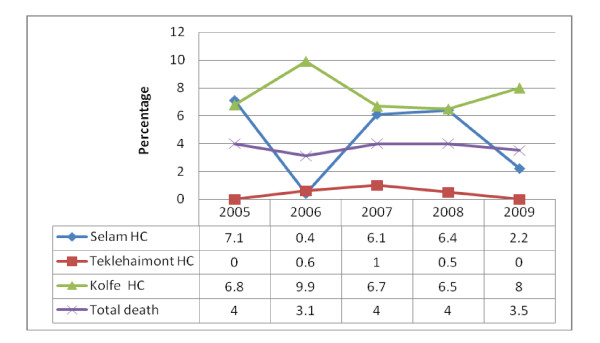
**Trend of TB mortality across the health center in Addis Ababa, March 2010**.

**Figure 3:**
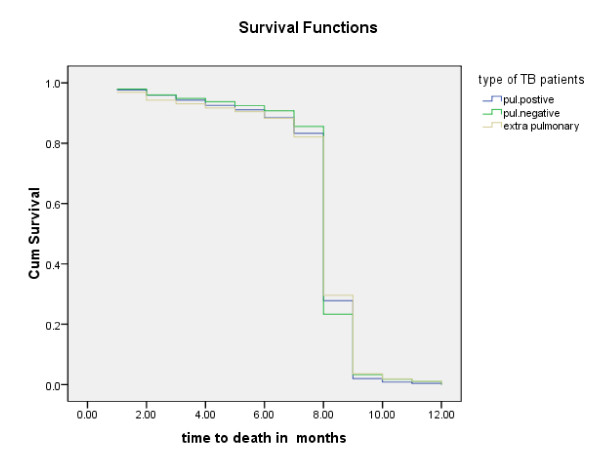
**Survival curve of pulmonary positive, pulmonary negative and extra pulmonary registered TB patients in Addis Ababa, Ethiopia, March 2010**.

Of the 6,450 registered patients, 6198(96.3%) survived the entire follow-up period. Time until an event (death, lost to follow up, transcestion due to treatment completion) for Person-years follow-up was 3753.67. The general mortality rate per 100 PYO was 6.3/100(6.3%) per annum for the cohort. The trend of TB mortality is being gradually showing decreasing in pattern as displayed in Figure 2. As depicted in Figure 3, more than 75% death occurred within eight month of treatment initiation. In this study, the proportion of death from pulmonary positives, pulmonary negatives and extra pulmonary TB patients were 2.7%, 3.6%, and 4.3%, respectively. Patient age, weight at initiation of anti-TB treatment, patient category, year of enrollment and treatment center were significantly associated with time to death (p < 0.05). TB patients weighting more than 34 kg at initiation of anti-TB treatment were 11.5% less likely to die [AHR = 0.899 with 95%CI of 0.804 to 0.973] compared with those weighting less than 34 kg. TB patients treated in Selam HC were 1.5 times [AHR = 1.498 CI at 95% (1.389- 1.616)] more likely to die compared to those treated in Teklehaimont HC. Compared to the years 1996-1997 the risk of death was significantly higher during 1997-1998 [AHR = 1.175 CI at 95% (1.047-1.320)], 1998-1999 [AHR = 1.207 CI at 95% (1.075-1.355), 2000-2001 [AHR = 1.137 CI at 95% (1.012-1.278)]. Moreover retreated TB patients were 1.74 times [AHR = 1.74 CI at 95% (1.439-2.096)] more likely to die compared to new Tb patients (Table 3).

**Table 3:** Results from multivariable Cox's proportional hazard model of risk factors for death the during treatment period, Addis Ababa, Ethiopia, March 2010.

Variables	Death status of the registered TB patients		Adjusted Hazard Ratio (AHR) (95% CI)	P-value
	alive (n; %)	died (n; %)		
**Treatment center**				0.000
Teklehaimont HC	2919(99.5)	16(0.5)	1.00	
Selam HC	947(95.8)	42(4.2)	1.50(1.40-1.60)	
Kolfe HC	2170(92.4)	178(7.6)	1.05(1.00-1.12)	
**Year of treatment**				0.001
July 2004 - June 2005	403(96.0)	17(4.0)	1.00	
July 2005 - June 2006	1388(96.8)	46(3.2)	1.18(1.05-1.32)	
July 2006 - June 2007	1520(95.8)	66(4.2)	1.21(1.078-1.36)	
July 2007 - June 2008	1341(96.0)	56(4.0)	1.06(0.95-1.19)	
July 2008 - June 2009	1384(96.4)	51(3.6)	1.14(1.01-1.28)	
**Patients category**				0.000
New	5373(96.4)	200(3.6)	1.00	
Retreated	663(94.8)	36(5.2)	1.74(1.439-2.096)	
**Body weight at initial stage of treatment**				0.012
weight < 34 kg	462(95.1)	24(4.9)	1.00	
weight ≥ 35 kg	5447(96.5)	197(3.5)	0.89(0.80-0.97)	

## Discussion

WHO defines TB mortality as the number of TB cases dying during treatment, regardless of the cause [13]. The mortality in our study was higher than in china [14] but lower than in those reported in previous studies including 24% in Baltimore City, USA [15], 14% in Vaud County, Switzerland [16] and similar to the mortality found in Addis (4%) and to the National (3%) [12]. The Mean and median duration from the onset of treatment to death in the current study were similar to a study conducted in south India, where by 94-97% of patients were surviving at the end of seven months of anti-TB treatment, irrespective of TB categories [17]. A study conducted in Chennai city [18] showed less survival rate (91%) compared to our study. This difference may be due to long follow up period in Chennai study and different number of study subjects. In other studies, 6% of the 676 patients died during the course of treatment; all patients who died had pulmonary TB [19]. Of the 39 patients who died, 65.0% died within 2 months of the start of treatment [19] and in other study, the survival probabilities were 97.5%, after the first month of treatment, 96% after the 3 months, 95% after 6 months and 93% after 1 year [20]. Unlike to our study, other studies have also found that a significant proportion of patients died in the early stages of treatment [21,22]. Early mortality reflects advanced disease and could be attributed to delayed treatment and late diagnosis [22,23]. In Vietnam study, similar to our finding, among 27 deceased patients the median time interval from treatment initiation to death was 8 months (IQR 5 to 11 months) [24]. Other studies demonstrated high risk of death during the first 2 months of anti-tuberculosis therapy [25-27]. Other study similar to our study showed the risk of mortality increased substantially among all subgroups from the second six months to the final eight months; hence it is likely that these deaths were due to drug sensitive or drug-resistant relapse [18].

In our study, general mortality rate per 100 PYO was 6.3/100(6.3%) per annum for the cohort which is similar to other studies. The general mortality rate for rural south India cohort was 61.0/1000 person-years or 6.1% per annum[28], which was not different from the mortality rate of 6% per annum reported for the Chennai urban cohort for the year 2000[18] but higher than a study in southern Ethiopia which shows mortality per 100 PYO was 2.5% per annum [29].

In our study baseline body weight was significantly associated with higher death. Similar finding was observed in south India, in which body weight at initiation of anti-TB treatment (<35 kgs) was a significant risk factors of death during anti-tuberculosis treatment period [17,19]. Other study also reported that among persons who were underweight at diagnosis, weight gain of 5% or less after two months of treatment was associated with an increased risk of relapse [30]. Similarly, low BMI was significant risk factor for mortality in patients with miliary tuberculosis (MTB) and lower BMI was found in patients who were died [31]. It may be assumed that malnutrition would be a clinical finding reflecting the severity of pulmonary TB (PTB) and that the determinant of in-hospital mortality would be due to PTB severity rather than malnutrition itself [32]. Underweight was associated with a 10-fold increase in TB mortality [33].

In our study treatment category was associated with higher death particularly previously treated patients. The characteristics of the TB patients associated with mortality during anti-TB treatment was being in retreatment case [34].Previously reported risk factors of mortality among tuberculosis patients include advanced human immunodeficiency virus (HIV) infection [35,36], multidrug resistance [36-38], and irregular treatment [39]. Similarly, multidrug resistance may explain the high combined failure and death rate of 55% among patients with transfer-out [24]. But in one study the mortality amongst the patients with previously treated TB was not statistically different [40]. This might be due to drug resistance cases at hospital level whereby most patients transferred or associated immune suppression at the beginning of the treatment and HIV prevalence may therefore be higher among patients in retreatment cases compared to patients with reported treatment success or these patients may not recover during treatment and may be transferred without well established referral system.

Our study is not without limitations. The data were extracted from medical records of those already visited and registered at the respective health facilities; it may be subjected to selection bias. In many tuberculosis patients, multiple causes of death may act simultaneously, so the cause of death may not be determined accurately [41,42]. In Norway this misclassification was shown to be considerable [43]. In the present study, the analyses included all deaths, irrespective of the cause of death, so misclassification of the cause of death might not have major influence on the results and as the nature of secondary data, some of the data are incomplete to gather the necessary variables such as HIV infection, patient's compliance, use of Directed Observed Treatment, use of ART, CPT and other prophylaxis, co-morbidity, drug resistance pattern. It is possible that some patients hidden their history of previous anti-tuberculosis treatment at the time of diagnosis and were inappropriately classified under different category. Despite the above limitations, this study was conducted at health centers that represent different level of potential health service coverage in sub-cities, which in turn reflect the TB patient under DOTS program at primary health care level in Addis Ababa and inclusion of several sites for comparison, and to get higher sample size to elucidate on the matter.

## Conclusion

Higher death rate was noted in patients with low baseline weights and patients who are previously treated cases. Additionally, the death rate was varied across year of enrollment. Therefore, reinforcing the existing DOTS program and nutritional support for underweight patients for better clinical and treatment outcome. Strengthen the recording system for completeness of the registry which is a common problem in practice and conducting further study with different design and area is needed to assess the TB mortality situation.

## Competing interests

The authors declare that they have no competing interests.

## Authors' contributions

BG conceived and designed the study and collected data in the field, performed analysis, Interpretation of data, and draft the manuscript. GA assisted with the design, interpretation of data and the critical review of the manuscript. SB, GM participated in design and performed analysis, interpretation of data, helped in drafting the manuscript and critically reviewed the manuscript. All authors and read and approved the final manuscript. All authors participated in critical appraisal and revision of the manuscript.

## Pre-publication history

The pre-publication history for this paper can be accessed here:

http://www.biomedcentral.com/1471-2334/11/127/prepub
